# Beyond the Bloodstream: MSSA Bacteremia with Severe Musculoskeletal Involvement

**DOI:** 10.51894/001c.166015

**Published:** 2026-07-31

**Authors:** Pooja Shah, Mikhail Y. Salnikov, Zohaib Khan, Abdallah Almawazreh, Mohamed Siddique

**Affiliations:** 1 DMC - Sinai Grace

## 122

### Introduction

We present a rare case of Methicillin-Susceptible Staphylococcus aureus (MSSA) bacteremia with widespread musculoskeletal involvement in a patient with complex infectious history.

### Case description

A 57-year-old male with a past history of bilateral septic knee arthritis, injection drug use, diabetes, hypertension, chronic pancreatitis, and MSSA bacteremia presented to the emergency department with complaints of right hip, right neck, and abdominal pain for 1 week.

On presentation, he was hypertensive and afebrile. On evaluation, he endorsed mild tenderness to palpation at the right cervical paraspinal muscles and right trapezius. Laboratory evaluation revealed leukocytosis, hyponatremia, hypokalemia, and an acute kidney injury. CT abdomen/pelvis showed right hip septic arthritis with destruction of the femoral head and acetabulum, discitis-osteomyelitis of T11-T12 and L4-L5 with right perivertebral and epidural abscesses, and right greater than left iliopsoas pyomyositis.

MRI of the cervical spine showed multilevel central canal stenosis extending from C3-C4 through C6-C7. C5-C6 featured central disc ossific complex and significant central canal stenosis and moderate to severe bilateral neural foraminal stenosis. MRI of the thoracic and lumbar spines showed discitis/osteomyelitis involving T11-T12 and L4-L5 levels. An epidural abscess extended from the L4-L5 interspace to L3-L4 with associated central canal stenosis. There was also a complex fluid collection in the right iliopsoas and right paraspinous soft tissues.

Management was initiated with empiric antibiotic therapy, followed by extensive infectious, cardiac, and orthopedic workup. Neurosurgery and orthopedic surgery both determined no surgical intervention required given improvement on IV antibiotics. Initial blood cultures grew Staphylococcus aureus, which was treated with intravenous nafcillin for 2 weeks followed by 2-week course of oral cephalexin. Repeat blood cultures 4 weeks later, remained negative for 48 hours. A TTE showed no evidence of endocarditis. The patient was discharged following 6 weeks of inpatient care for primary infection and other comorbidities and was advised to follow up with orthopedic surgery.

### Discussion

This case highlights an advanced presentation of MSSA infection with manifestations of soft tissue infection and myeloradiculopathy. Clinicians should be aware of the extent to which MSSA can disseminate to cause further destruction where seeding of bacteria can occur.

